# A low frequency damaging *SORCS2* variant identified in a family with ADHD compromises receptor stability and quenches activity

**DOI:** 10.1038/s41380-025-03242-3

**Published:** 2025-09-18

**Authors:** Mathias Kaas, Sarah Broholt Dinesen, Ole Ahlgreen, Peder Madsen, Simon Mølgaard, Anders Dalby, Camilla Gustafsen, Ditte Olsen, Jinjie Duan, Joachim Vilstrup, Jonas Lende, Sanne Nordestgaard, Tetyana Zayats, Per Morten Knappskog, Stefan Johansson, Gesche Neckelmann, Barbara Franke, Søren Thirup, Anders Børglum, Andreas Reif, Christian Vægter, Ditte Demontis, Jan Haavik, Simon Glerup, Sune Skeldal

**Affiliations:** 1 Department of Biomedicine, Aarhus University, Aarhus, Denmark; 2 Teitur Trophics, Aarhus N, Denmark; 3 Department of Molecular Biology and Genetics, Aarhus University, Aarhus, Denmark; 4 Broad Institute of MIT and Harvard, Cambridge, MA USA; 5 Department of Biomedicine, University of Bergen, Bergen, Norway; 6 Center for Medical Genetics and Molecular Medicine, Haukeland University Hospital, Bergen, Norway; 7 Department of Clinical Science, University of Bergen, Bergen, Norway; 8 Department of Radiology, Haukeland University Hospital, Bergen, Norway; 9 Departments of Medical Neuroscience and Human Genetics, Donders Institute for Brain, Cognition and Behaviour, Radboud University Medical Center, Nijmegen, The Netherlands; 10 Department of Psychiatry, Psychosomatic Medicine and Psychotherapy, University of Frankfurt, Frankfurt, Germany; 11 Division of Psychiatry, Haukeland University Hospital, Bergen, Norway

**Keywords:** Neuroscience, Molecular biology, ADHD

## Abstract

Attention-deficit/hyperactivity disorder (ADHD) is a common neurodevelopmental disorder affecting 5% of children and 2.5% of adults worldwide. ADHD is considered a polygenic disorder caused by a combination of both common and rare risk variants, each with low individual effect size. The Vps10p domain receptor SorCS2 is involved in neuronal development and synaptic plasticity by modulating brain-derived neurotrophic factor (BDNF) signaling. We here describe the identification and characterization of a heterozygous damaging variant in the *SORCS2* gene found in two members of a family with persistent ADHD. The *SORCS2* variant results in an arginine to tryptophan substitution in the 10CC region of the extracellular Vps10p domain, leading to aberrant posttranslational receptor processing, subcellular localization and ligand binding. Furthermore, the variant abrogates BDNF signaling in a dominant negative manner. Biochemical analysis of additional rare missense variants from ADHD cohorts suggested that SorCS2 structural stability and function is susceptible to such variation in the Vps10p domain. Our findings provide insights into how low frequency damaging variants in *SORCS2* may contribute to the risk of ADHD.

## Introduction

ADHD is a neurodevelopmental disorder characterized by motor hyperactivity, inattention and impulsiveness - all to an extent, which causes significant impairment for those affected. ADHD often runs in families and has a strong genetic component [1]. Based on family and twin studies, the heritability is estimated to be 74% [2]. In up to 2/3 of children with ADHD the disorder persists into adulthood [3]. ADHD is considered highly polygenic in nature involving more than 7000 common variants each with small effect size and under the influence of environmental factors [4].

SorCS2 belongs to the Vps10p domain receptor family also encompassing sortilin, SorLA and SorCS1 and -3. These receptors all have critical roles in neuronal function [5–12] and are genetically implicated in several neurological disorders [4, 13–20]. SorCS2 is involved in signaling by brain-derived neurotrophic factor (BDNF) [5, 6, 21, 22], plays a critical role in guiding dopaminergic axons during embryonic development [5] and is essential for synaptic plasticity in the postnatal hippocampus [6]. *Sorcs2* knockout mice are characterized by distinct behavioral abnormalities including hyperactivity, reduced anxiety, altered alcohol-seeking behavior, reduced prepulse inhibition as well as deficient learning and memory [5, 6, 23, 24]. Strikingly, the hyperactivity of *Sorcs2* knockout mice in an open field paradigm is normalized following the administration of amphetamine [5]. Such paradoxical response to central stimulants is the clinical hallmark of ADHD in humans and renders *SORCS2* an attractive candidate risk gene in ADHD. In support of this, one study identified a genetic variant in *SORCS2* that showed suggestive levels of association with persistent ADHD [20]. Furthermore, *SORCS2* methylation is proposed to be involved with grey matter volume in the precentral and posterior orbital gyri and emotional behaviour problems in children with ADHD in a study of monozygotic twins [25]. *SORCS3* has been identified as a common risk gene in several psychiatric disorders including ADHD [26], where also rare deleterious *SORCS3* variants were associated at nominal significance [4], further supporting the role of SorCS receptors in psychiatric pathobiology.

We here describe a low frequency damaging missense variant in *SORCS2* found in a family with individuals diagnosed with persistent ADHD. The variant disrupts biochemical and trafficking properties of SorCS2 and abolishes SorCS2-dependent BDNF-signaling. We further characterized additional low frequency *SORCS2* missense variants from individuals suffering from ADHD. Taken together, our results demonstrate how low frequency *SORCS2* missense variants from ADHD patients affect SorCS2 biology and suggest that SorCS2 perturbations could contribute to the etiology of persistent ADHD.

## Results

### The *SORCS2* variant substitutes a highly conserved arginine to a tryptophan

**Fig. 1:**
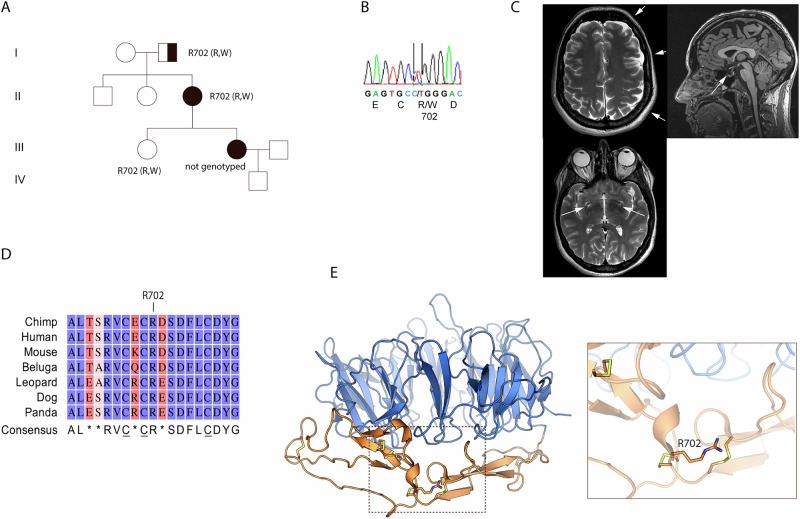
Identification of the R702W SorCS2 variant. **A** family pedigree showing status of SorCS2 R702W heterogenous variant. Squares and circles represent males and females, respectively. Black symbol indicates individuals diagnosed with persistent ADHD while half-blackened symbol indicates a non-assessed individual with signs of ADHD. Roman numerals indicate the generation. **B** DNA sequencing profile of an affected family member showing the heterozygous single base substitution C to T at position 2104 in exon 16 of *SORCS2*. **C** T1-weighted MRI scan of second-generation individual diagnosed with ADHD and carrying the R702W variant. Arrows indicate pronounced cortical relief (upper left), wide partially empty sella (upper right), and perivascular spaces (bottom) in 3 T. **D** amino acid alignment between various species of the sequence surrounding arginine 702. Blue and red colors indicate highly conserved residues and non-conserved residues, respectively. Light red indicates modestly conserved residues. Consensus sequence is indicated and disulfide forming cysteines are underscored. **E** SorCS2 Vps10p 3D structure (PDB 6FG9) with side chain of R702 and surrounding disulfide bonds (yellow) depicted in the 10CC module (orange). The β-propeller is shown in blue.

As part of a nation-wide study investigating clinical features and risk factors among Norwegian individuals with ADHD, control persons and families with ADHD, we performed exome sequencing in 21 families that had reported multiple cases of persistent ADHD [27]. In a family reporting three family members with ADHD, we found three individuals who were heterozygous for the variant (4-7716081-C-T) in exon 16 of *SORCS2*, resulting in the substitution of an arginine to a tryptophan at amino acid position 702 (R702W) (Fig. 1A and B). Two of the three R702W carriers were diagnosed with ADHD, and one was not. We had limited access to clinical information from four generations from this family, which however suggested an increased burden of ADHD in the family, but no clear pattern of segregation with the R702W variant suggestive of Mendelian inheritance. Brain magnetic resonance imaging (MRI) data was obtained from 81 participants in the Norwegian adult ADHD project [28], including the second-generation R702W carrier from this family diagnosed with ADHD. Overall, the MRI scan showed no pathological abnormalities. As shown in Fig. 1C, T1 weighed brain imaging showed normally configurated and sized inner liquor spaces. There was a certain asymmetry in the outer liquor space with a slightly more pronounced cortical relief in the left hemisphere. The basal ganglia were unremarkable, as were the cerebellopontine angles and the craniocervical junction. Partially empty sella and a few wide perivascular spaces were also observed (Fig. 1C).

### R702W changes SorCS2 trafficking, localization and shedding

The R702 residue is highly conserved among species (Fig. 1D) and the R702W variant is reported 39 times in GnomAD [29], rendering an allele frequency of 0.000025. SorCS2 displays global homology with SorCS1 and SorCS3 and consists of an N-terminal Vps10p domain followed by a two fibronectin like domains, a domain with similar fold as found in RNA-binding proteins, a transmembrane region, and a 60 amino acid C-terminal cytoplasmic tail. R702 is located in the 10CC module of the SorCS2 Vps10p domain; a structurally important unit encompassing five highly conserved disulfides bridges, critical for proper folding of the entire domain (Fig. 1E). We therefore cloned and expressed the R702W SorCS2 variant and compared its processing, subcellular localization and stability to that of wildtype (WT) SorCS2.

**Fig. 2:**
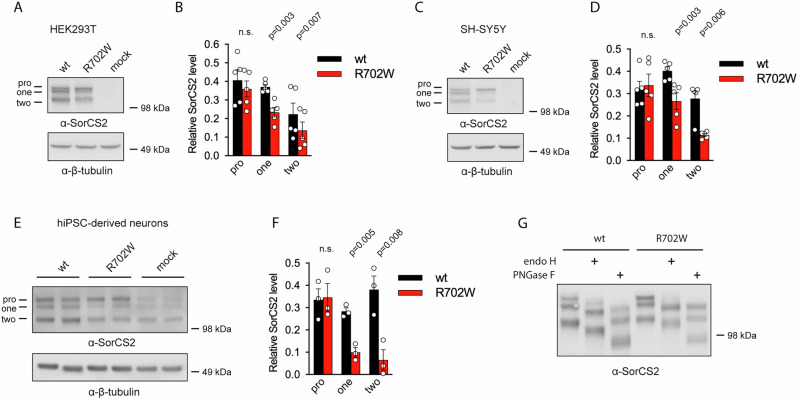
The R702W substitution affects relative levels of SorCS2 processed forms and glycosylation pattern. **A** and **C** representative western blot (n = 5) showing reduced levels of the one and two chain SorCS2 R702W in HEK293T and SH-SY5Y cells. **B** and **D** quantification of A and C. **E** levels of one- and two-chain SorCS2 R702W are reduced in transfected human iPSC-derived neurons (n = 3, experiments performed in technical duplicates). Of note, these cells express endogenous SorCS2 as seen with the mock transfected cells. **F** quantification of E shown with endogenous level of SorCS2 subtracted. **G** western blot showing that all N-linked oligosaccharides of the proform of SorCS2 R702W can be fully removed with endo H treatment suggesting that the proform of SorCS2 R702W contains mainly high-mannose N-linked oligosaccharides (n = 3).

**Fig. 3:**
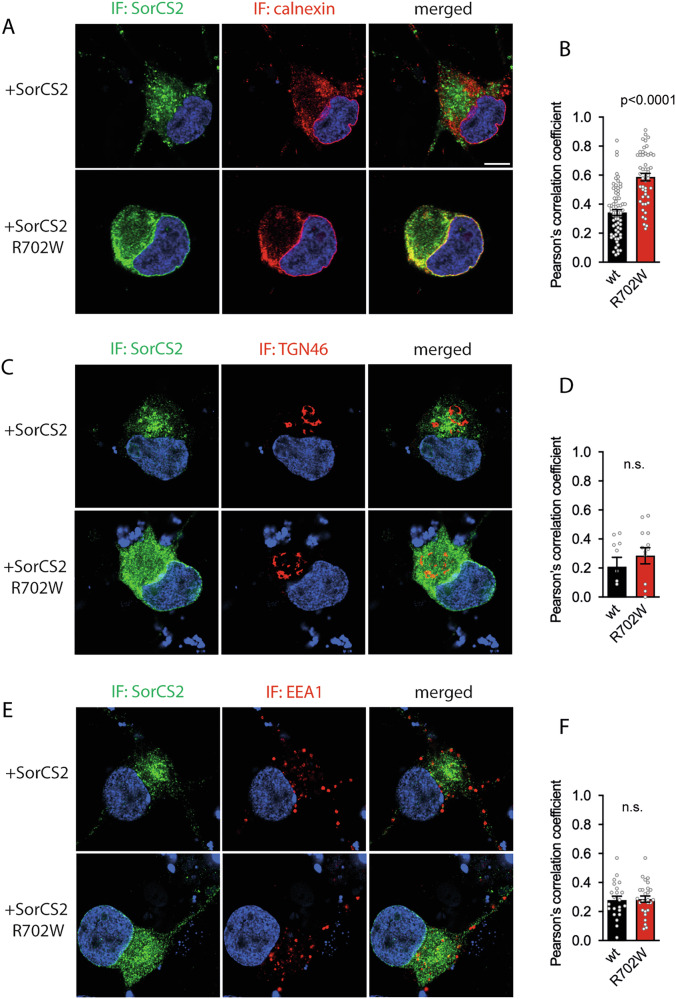
The R702W substitution causes aberrant subcellular localization of SorCS2. **A,** **C** and **E** coimmunostaining of SorCS2 variants and calnexin (A), TGN46 (C), and EEA1 (E) in transfected iPSC-derived neurons. Hoechst staining is shown in blue. **B D,** **F** quantification of A, C, and E shown as Pearson’s correlation coefficient for SorCS2 variants and respective subcellular markers. Results are shown as mean ± SEM, n = 69 (SorCS2 WT, calnexin), n = 49 (SorCS2 R702W, calnexin), n = 10 (SorCS2 WT, TGN46), n = 13 (SorCS2 R702W, TGN46), n = 22 (SorCS2 WT, EEA1), n = 27 (SorCS2 R702W, EEA1), statistical analysis by unpaired two tailed Student’s *t* test, IF, immunofluorescence. Scale bar is 5 μm.

**Fig. 4:**
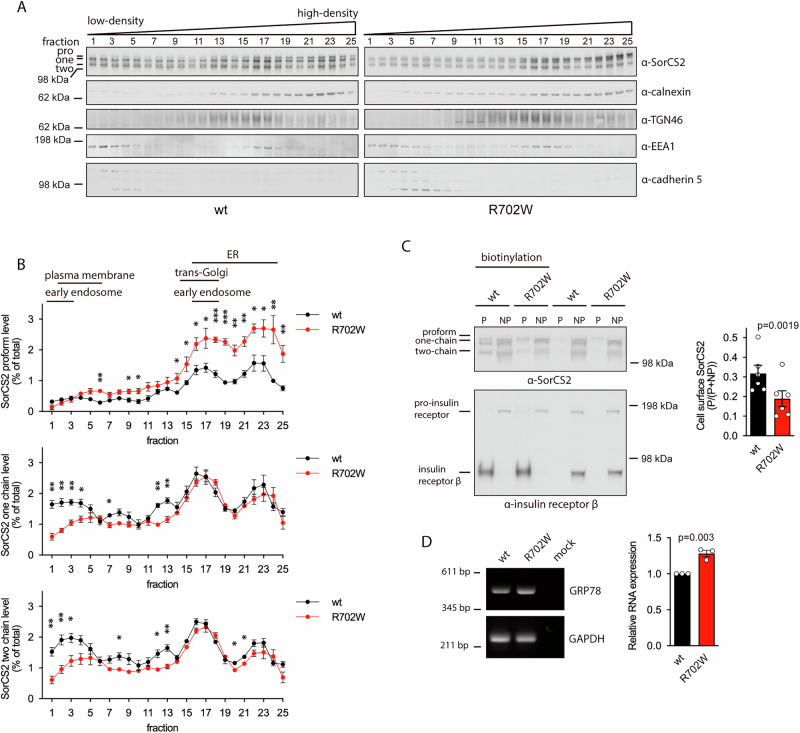
The R702W substitution changes the subcellular distribution of SorCS2 processed isoforms. **A** representative western blot of sucrose gradient centrifugation of HEK293T cells transfected with SorCS2 WT or R702W. **B** quantification of A shows the level of SorCS2 processed forms in each fraction as percentage to total SorCS2 for WT or R702W SorCS2 (mean ± SEM, n = 3–4, statistical analysis by paired two tailed Student’s *t* test, *=p ≤ 0.05, **=p ≤ 0.01, ***=p ≤ 0.001). **C** surface proteins in HEK293T cells transfected with WT or R702W SorCS2 were biotinylated on ice and biotinylated SorCS2 precipitated with streptavidin (P). Non-precipitable SorCS2 (NP) represents intracellular SorCS2. Whereas a fraction of SorCS2 WT localizes to the cell surface, SorCS2 R702W is mainly found intracellularly. Precipitation of biotinylated endogenous insulin receptor (lower western blot) demonstrates that samples are biotinylated and precipitated equally well. Shown is mean ± SEM (n = 6). Statistical analysis by paired two tailed Student’s *t* test. **D** semi-quantitative RT-PCR showing increased expression of GRP78 in HEK293T cells when transfected with SorCS2 R702W relative to WT. Data shown as mean ± SEM (n = 3). Data analyzed by unpaired two tailed Student’s *t* test.

Tissue- and cell-type specific proteolytic processing of SorCS2 generates three variants, a single-chain proform, a single-chain mature receptor and a two-chain mature receptor evident from the presence of three distinct SorCS2 bands visualized by western blotting using antibodies against SorCS2 extracellular domain [5]. Correct processing is of biological importance as one- and two-chain SorCS2 display different functions [5]. In contrast to SorCS2 WT, SorCS2 R702W had reduced levels of one- and two-chain forms when analyzed by western blotting of lysates from transfected HEK293T and SH-SY5Y cells (Fig. 2A–D, Supplementary Fig. 1A and B). Similarly, human iPSC-derived neurons transfected with SorCS2 R702W had markedly less one- and two-chain forms when compared to neurons transfected with WT SorCS2 (Fig. 2E and F, Supplementary Fig. 1C). Expression of the SorCS2 proform was seemingly unaltered by the R702W mutation in all cell lines (Fig. 2A–F). The altered gel band pattern of the R702W variant was not due to changes in glycosylation as treatment with N-linked deglycosylation enzyme PNGase F did not change the ratio between SorCS2 R702W processing isoforms (Fig. 2G, Supplementary Fig. 1D). Interestingly, the proform of SorCS2 R702W contained mainly high-mannose oligosaccharides as treatment with high-mannose deglycosylation enzyme endo H reduced its molecular weight similar to treatment with the N-linked deglycosylation enzyme PNGase F (Fig. 2G). Immunofluorescent staining of transfected iPSC-derived neurons further revealed that the R702W SorCS2 variant was localized to subcellular compartments distributed throughout the cell in contrast to the WT variant, which was observed in distinct paranuclear vesicular structures. By using Pearson’s correlation coefficient, expression of SorCS2 R702W was significantly more correlated with the endoplasmic reticulum marker protein calnexin compared to SorCS2 WT (Fig. 3A and B). In contrast, correlation of SorCS2 R702W with the trans-Golgi marker TGN46 and the early endosome marker EEA1 did not differ from SorCS2 WT (Fig. 3C–F). However, quantitative analysis using Manders’ overlap coefficient M2 did suggest increased colocalization of SorCS2 R702W with TGN46 and EEA1 relative to SorCS2 WT (Supplementary Fig. 2A–C). To corroborate these findings, we analyzed the bulk subcellular localization of SorCS2 variants by separating cellular structures according to their density using sucrose gradient centrifugation. Quantification of the SorCS2 WT processing isoforms revealed that the SorCS2 proform was mainly found in two large peaks in higher-density fractions whereas the single- and two-chain forms were more abundant in lower-density fractions including a large peak in the lowest density fractions (Fig. 4A and B). Interestingly, the level of SorCS2 R702W was significantly reduced in this low-density peak and the R702W proform was markedly enhanced at the highest density fractions. To interpret the observed fractionation pattern, we compared the localization of R702W SorCS2 to that of calnexin, TGN46, EEA1 and the cell surface marker cadherin-5 (Fig. 4A). As such, cadherin-5 was found in the fractions of lowest density and calnexin in the two peaks of high-density also encompassing the majority of the R702W SorCS2 variant. TGN46 was found in fractions of intermediary density partly overlapping with calnexin. Thus, the presence of R702W appears to arrest SorCS2 in the early biosynthetic pathway and reduce its cell surface localization. We therefore performed cell surface biotinylation experiments and confirmed the reduced localization of SorCS2 R702W at the cell surface (Fig. 4C).

**Fig. 5:**
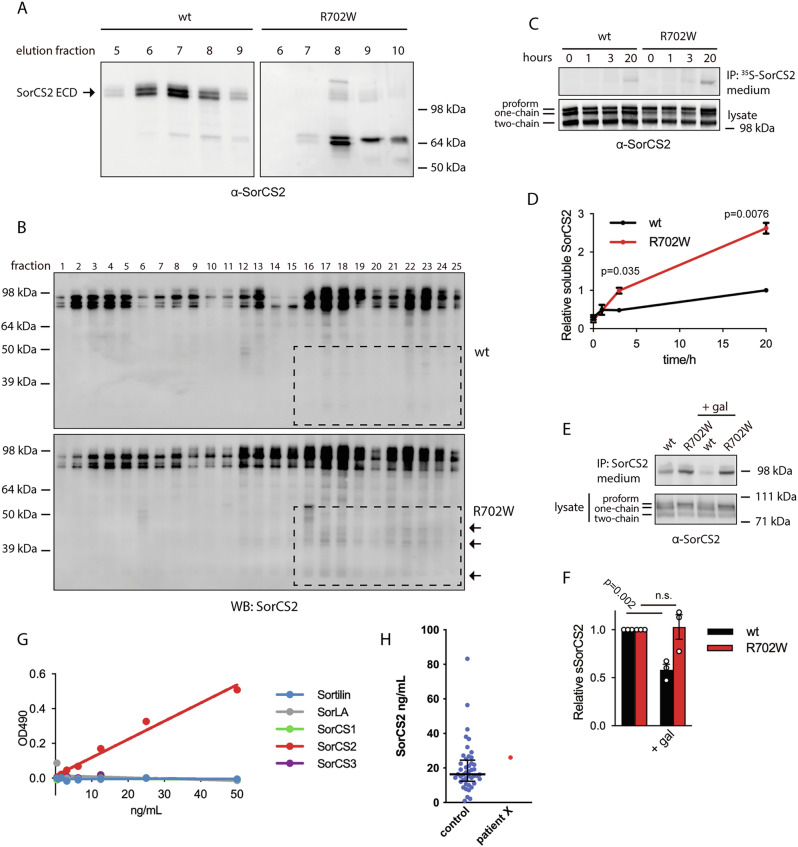
R702W SorCS2 is susceptible to aberrant proteolytic cleavage. **A** western blot of elution profile from immunoaffinity chromatography purification of WT and R702W SorCS2 ECD (n = 1). **B** sucrose gradient centrifugation of HEK293T cells transfected with WT or R702W SorCS2 (n = 3). Multiple fragments of R702W SorCS2 in high-density fractions are indicated. **C** pulse-chase experiment showing increased release of R702W SorCS2 ECD. Transfected HEK293T cells were biolabeled and SorCS2 immunoprecipitated from culture supernatant at indicated time points and subjected to SDS-PAGE and autoradiography. Western blot (n = 3) of stead-state level of SorCS2 in cells is also shown. **D** quantification of C. Values are normalized to WT SorCS2 at 20 h and shown as mean ± SEM. Data analyzed by unpaired two tailed Student’s *t* test. **E** representative western blot of released SorCS2 ECD immunoprecipitated from culture supernatant of transfected HEK293T cells in the presence or absence of 30 µM galardin (gal). **F** quantification of E. Shown is mean ± SEM (n = 3). Data analyzed by unpaired two tailed Student’s *t* test. **G** and **H** a SorCS2 specific ELISA was established (G) in order to measure serum levels of SorCS2 ECD in a control group and a member of the affected family (patient X) (n = 1). Median with interquartile range is shown for the control group.

To further study the apparent ER retention of SorCS2 R702W, we assessed expression of the ER stress marker GRP78 in transfected cells by semi-quantitative PCR. The data was suggestive of an increased expression of GRP78 in cells transfected with SorCS2 R702W indicating increased activity of the ER quality control system as a measure to cope with misfolded SorCS2 R702W (Fig. 4D). To characterize potential perturbations of stability and ligand properties caused by the R702W variant, we cloned and expressed the extracellular domain (ECD) of WT and R702W SorCS2, which was subsequently purified by immunoaffinity chromatography. Unfortunately, the R702W ECD was degraded into multiple fragments during the purification process, suggesting compromised stability and increased susceptibility to proteolytic degradation (Fig. 5A). Fragments from degraded full-length SorCS2 R702W expressed by HEK293T cells were also apparent in the high-density fractions of sucrose gradient centrifugations suggesting that degradation takes place early in the biosynthetic pathway (Fig. 5B). To further monitor the processing and turnover of the full-length receptor variants, we metabolically labeled transfected HEK293T cells with ^35^S-methionine and -cysteine and performed pulse-chase analysis. Interestingly, we observed markedly increased release of ECD SorCS2 R702W to the medium compared to WT suggesting increased shedding of R702W SorCS2 (Fig. 5C and D) [30]. Accordingly, shedding of SorCS2 WT was reduced by the broad-spectrum metalloproteinase inhibitor galardin. However, liberation of SorCS2 R702W ECD was completely insensitive to metalloproteinase inhibition, suggesting that the R702W variant renders SorCS2 a substrate for other classes of cell proteinases (Fig. 5E and F). To understand how levels of SorCS2 ECD in extracellular fluids was influenced by the presence of the R702W substitution, we developed a SorCS2-specific ELISA and measured SorCS2 in 36 control individuals and the second-generation ADHD-diagnosed individual of the affected family (Fig. 5G and H). The median SorCS2 level of controls was 16.3 ng/ml with values spanning from 0.92–83.3 ng/ml. Of note, the affected family member had a value of 26.1 ng/ml serum SorCS2.

### SorCS2 R702W forms heterodimers with SorCS2 WT and quenches BDNF signaling

**Fig. 6:**
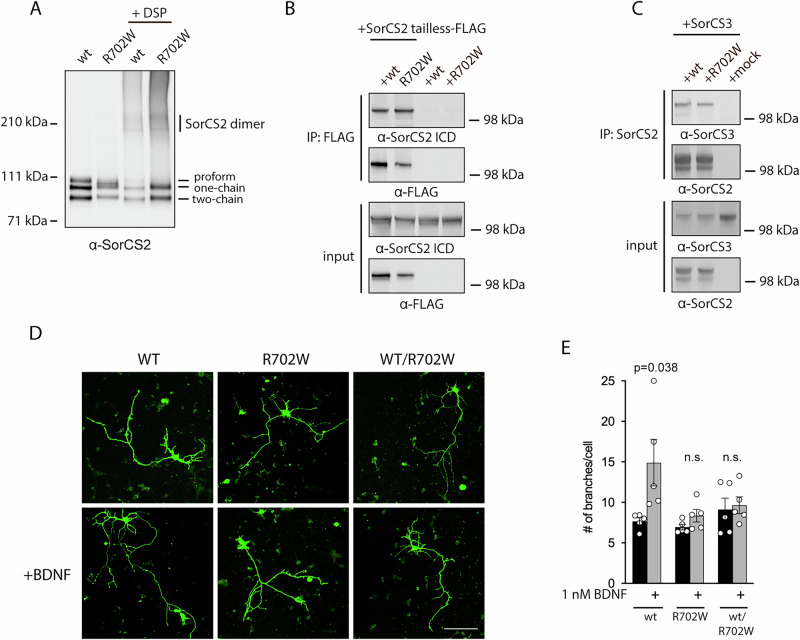
The R702W variant forms dimers and quenches SorCS2 WT activity. **A** western blot showing homodimerization of both WT and R702W SorCS2 in transfected HEK293T cells after cross-linking with DSP (n = 3). **B** R702W SorCS2 can be co-immunoprecipitated with WT SorCS2 tailless in HEK293T cells as assessed with an antibody recognizing SorCS2 intracellular domain (ICD) (n = 3). **C** western blot showing that SorCS3 co-immunoprecipitates equally well with WT and R702W SorCS2 in transfected HEK293T cells (n = 3). **D** *Sorcs2* knockout hippocampal neurons were transfected with the indicated constructs and GFP and neuronal branching assessed in the presence or absence of 1 nM BDNF for 72 h. Scale bar is 100 μm. **E** quantification of D. Values are shown as mean ± SEM (n = 5). Statistical analysis by unpaired two tailed Student’s *t* test.

A key feature of transmembrane type I receptors engaged in cellular signaling is their ability to form multimeric receptor complexes. SorCS2 is well described to form homodimers in solution and as a transmembrane receptor in cells [31, 32]. To study the capacity of full-length SorCS2 R702W to form homodimers we analyzed the migration pattern of SorCS2 by non-reducing SDS-PAGE in the presence or absence of the protein cross-linker agent DSP. Like WT, cross-linking rendered SorCS2 R702W to migrate as a group of high-molecular weight bands corresponding to the size of a dimer (Fig. 6A) suggesting that SorCS2 R702W has retained its ability to form dimers. Additionally, SorCS2 receptor complexes were readily formed between WT and R702W SorCS2 as the latter can be co-precipitated with WT SorCS2 lacking its intracellular domain (SorCS2 tailless) (Fig. 6B).

As SorCS2 possesses the capacity to form heterodimeric receptor complexes with other SorCS receptors [32], the R702W variant may potentially interfere with other receptors of the family. We therefore studied complex formation between SorCS2 R702W and SorCS3 in cells by co-immunoprecipitation. Here, SorCS2 R702W was clearly able to form a receptor complex with SorCS3 (Fig. 6C and Supplementary Fig. 3).

Prompted by the apparent ability of SorCS2 R702W to form dimers with WT SorCS2, we sought to address whether SorCS2 R702W would perturb normal SorCS2 function. As a model system, we took advantage of a neuronal branching assay using *Sorcs2* knockout neurons as previously established [6]. Neurons were prepared from P0 pups and transfected at DIV1 with combinations of GFP and SorCS2 WT and/or SorCS2 R702W in the presence or absence of BDNF (1 nM), fixed at DIV4, and neuronal branching of GFP-positive neurons quantified. No BDNF-induced increase in neuronal complexity was observed in *Sorcs2* knockout neurons transfected with GFP alone as previously reported [6]. Interestingly, whereas co-transfection with SorCS2 WT induced a robust BDNF response in GFP-positive neurons, no such response was observed with SorCS2 R702W. Furthermore, when we performed combined transfections with WT and R702W encoding constructs, the presence of the R702W variant quenched the ability of SorCS2 WT to mediate BDNF activity, suggesting a dominant negative effect of R702W in this experimental setup (Fig. 6D and E).

### Analysis of additional low frequency missense *SORCS2* variants from ADHD cohorts

**Fig. 7:**
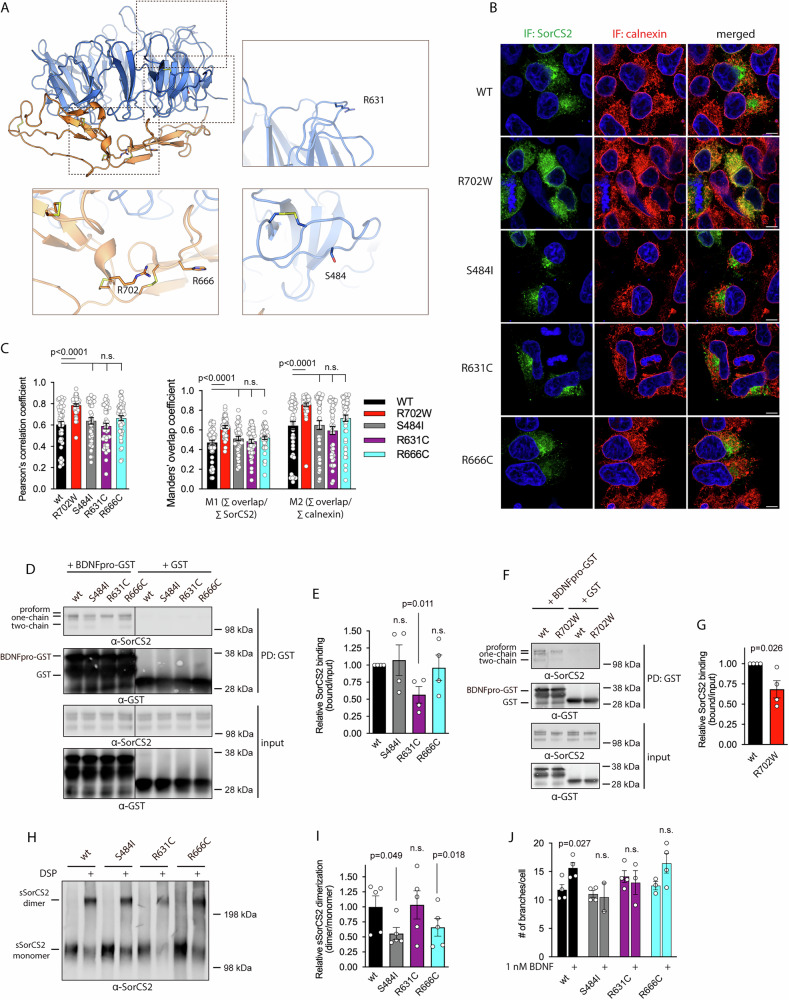
Low frequency SorCS2 missense variants hamper SorCS2 functions. **A** 3D structure of SorCS2 Vps10p domain (PDB 6FG9) with side chain of mutated amino acids in ADHD patients depicted. The structure depicts mouse SorCS2 and thus the R666 position is here shown as the mouse endogenous histidine. **B** coimmunostaining of SorCS2 variants and calnexin in transfected HEK293T cells. Hoechst staining is shown in blue. **C** quantification of B shown as Pearson’s correlation coefficient (left) and Manders’ overlap coefficient (right). Results are shown as mean ± SEM, n = 47 (SorCS2 WT), n = 46 (R702W), n = 42 (S484I), n = 46 (R631C), n = 48 (R666C), statistical analysis by unpaired two tailed Student’s *t* test, IF, immunofluorescence. Depicted scale bar is 5 µm. **D** western blot of pull downs of SorCS2 variants from transfected cells with BDNFpro-GST. **E** quantification of D shown as mean ± SEM (n = 4). Data analyzed by unpaired two tailed Student’s *t* test **F** western blot of pull down of R702W SorCS2 from transfected cells with BDNFpro-GST. **G** quantification of F shown as mean ± SEM (n = 4). Data analyzed by paired two tailed Student’s *t* test. **H** western blot showing cross-linking of dimers of SorCS2 ECD variants secreted by transfected CHO cells. **I** quantification of H. Shown as mean ± SEM (n = 5). Data analyzed by paired two tailed Student’s *t* test. **J** neuronal branching of hippocampal *Sorcs2* knockout neurons assessed after transfection with SorCS2 variants in the presence or absence of 1 nM BDNF. Values are shown as mean ± SEM (n = 2–4). Statistical analysis by unpaired two-tailed Student’s *t* test.

We further investigated whether low frequency damaging *SORCS2* variants might be found in other ADHD cohorts by studying two separate cohorts. The first was from an exome-wide scan using the Illumina Exome Bead Chip of 597 individuals with ADHD and 433 controls from Bergen, Norway [33] while the second cohort consisted of whole exome sequencing data of 1078 German and Dutch individuals with ADHD and 1738 controls (Demontis and Duan et al. unpublished). Together, 38 low frequency (ExAC allele count <0.01) *SORCS2* missense variants were observed among individuals with ADHD while 65 were observed in controls (Supplementary Table 1). We used SIFT [34], Polyphen-2 [35], CADD-PHRED [36], MutationTaster [37], AlphaMissense [38] and MPC [39] scores to estimate deleteriousness of the observed variants, which showed no significant overrepresentation of damaging variants in ADHD patients (p = 0.55 Fisher exact test). However, several variants observed were interesting from a structural and functional perspective and were studied further. We focused on SorCS2 S484I, R631C and R666C that represented one variant shared among patients and controls (S484I) and two patients only variants (R631C and R666C) which were located in the Vps10p domain near the NGF binding site or in close proximity to R702 (Fig. 7A). To assess the effect of selected substitutions on SorCS2 structural integrity, we first evaluated ER retention of SorCS2 by studying colocalization of SorCS2 variants with calnexin in transfected HEK293T cells. Whereas SorCS2 R702W clearly displayed increased colocalization with calnexin compared to SorCS2 WT, no such effect was observed with the other SorCS2 variants (Fig. 7B and C). To further investigate structural integrity of the Vps10p domain we next studied ligand binding properties of SorCS2 variants by pull down using the prodomain of proBDNF (BDNFpro), which was previously reported to bind SorCS2 extracellular domain with nanomolar range affinity. BDNFpro binding of SorCS2 R631C was reduced to an extent which seemingly exceeded the reduction of the R702W variant (Fig. 7D–G). As an approach to further study consequences of amino acid substitutions on Vps10p domain structural integrity, we studied the ability of SorCS2 variants to form homodimeric receptor complexes in the culture supernatant from cells transfected with SorCS2 ECD constructs. To this end, we first performed size-exclusion chromatography of SorCS2 WT ECD, which eluted with an apparent MW of approximately 515 kDa, indicating homo-oligomerization or a highly elongated structure (Supplementary Fig. 4A). In non-reducing SDS-PAGE, SorCS2 migrated as a single band, corresponding to a SorCS2 ECD monomer [5] However, upon treatment of the the sample with DSP, SorCS2 migrated as a single band corresponding to a SorCS2 ECD dimer (Supplementary Fig. 4B) suggesting that dimerization of SorCS2 ECD in solution indeed can be monitored using this experimental setup. All variants were secreted and migrated as a ~ 100 kDa band by non-reducing SDS-PAGE corresponding to a monomer. Upon cross-linking, SorCS2 variants migrated as a ~ 200 kDa band corresponding to a dimer, however only a fraction of SorCS2 S484I and R666C were able to form dimers suggesting that these substitutions compromise SorCS2 dimerization (Fig. 7H and I).

We finally studied the biological consequence of these variants in terms of BDNF activity of SorCS2. Like the R702W variant, all variants abrogated the ability of SorCS2 to rescue BDNF-induced response in transfected *Sorcs2* knockout neurons (Fig. 7J and Supplementary Fig. 4C). Hence, selected low-frequency *SORCS2* missense variants found in ADHD and control cohorts affect SorCS2 biochemical and biological properties in a similar manner as for the R702W variant.

## Discussion

The study of families with a history of neurological and psychiatric disorders has greatly improved our understanding of the genetics and molecular biology of the underlying disorders. For example, the Swedish variant in APP which was found to segregate with early onset Alzheimer’s disease in two large Swedish families [40] has significantly facilitated the understanding of Alzheimer’s disease. Similarly, the discoveries of disrupted in schizophrenia-1 (DISC1) by cloning of a chromosomal translocation that segregated with a spectrum of major mental illnesses in a Scottish family [41], and variants in *NLGN3* [42] and *SHANK3* [43] in families suffering from autism spectrum disorder, have made seminal contributions to the understanding of how malfunction of synaptic molecular networks underlies mental disorders.

ADHD results from a polygenic risk load of common variants each with low effect size and in around 1 out of 4 individuals with ADHD rare deleterious variants with a higher effect size also contributes to the risk [4, 44–46]. By exome sequencing of a Norwegian family suffering from persistent ADHD, we identified a rare heterozygous missense variant in *SORCS2* substituting an arginine to a tryptophan at position 702. As the number of family members was low, access to family members for sequencing limited and one carrier was not diagnosed with ADHD, it was impossible to establish a clear pattern of segregation for this variant, limiting the confidence with which we can associate the R702W variant with ADHD. The observed healthy carrier may indeed reflect that the R702W variant has incomplete penetrance and manifests itself only in combination with certain polygenic risk background and environmental exposure which are core features of ADHD pathogenesis [4]. Common variants in *SORCS2* have been associated with multiple traits and phenotypic markers related to ADHD, including, educational attainment, risk-taking behavior [47], plasma cholesterol and lipoprotein levels [48, 49] and type 2 diabetes mellitus [50]. Together with the known functions of SorCS2 in neurotrophic signaling and neuronal wiring, and the calming effect of amphetamine on the behavior of *Sorcs2* knockout mice [5, 51], this adds support for the notion that *SORCS2* perturbations could be involved in ADHD pathogenesis. Our analysis of additional *SORCS2* missense variation did not provide evidence of general contribution to ADHD. As more patient samples are being sequenced, it will be possible to explore the genotype-phenotype relationship of R702W and other *SORCS2* variants in further detail.

R702 is spatially located near two disulfide bonds within the 10CC module, a domain which interacts and stabilizes the ligand binding beta-propeller of the Vps10p domain [31]. As such, the 10CC module has a low content of secondary structure but obtains rigidity through five highly conserved disulfide bridges [31, 52, 53]. It is plausible that substitution of the arginine to a tryptophan interferes with disulfide bond formation in the 10CC module, which further compromises the structural integrity of the entire Vps10p domain. In line with this, SorCS2 R702W was mainly represented by its proform with a high content of high-mannose oligosaccharides suggesting that the proform of SorCS2 R702W does not pass the protein quality control system in the ER and therefore is not further posttranslationally processed. Indeed, the subcellular distribution of SorCS2 R702W showed a high degree of overlap with the ER marker protein calnexin and reduced level of processed forms at the cell surface. Reduction of cell surface localized SorCS2 R702W is likely further augmented by unspecific proteolytic cleavage of SorCS2 R702W at the cell surface in line with the observed increased liberation of SorCS2 R702W ECD in HEK293T cells. Alternatively, but not mutually exclusive, the enhanced release of SorCS2 R702W ECD could be caused by elevated proteolytic degradation and release from intracellular compartments. The apparent accumulation of SorCS2 R702W within the ER and reduced levels at the cell surface likely render SorCS2 unable to engage with its described signaling and sorting functions. Interestingly, a recent study assessing thousands of pathogenic missense variants showed that subcellular mislocalization was a common consequence across different diseases and genes [54], supporting the notion that R702W could be a pathogenic variant.

Among the other tested variants, BDNFpro binding was significantly impaired for R631C SorCS2. This is not surprising as R631 localizes to the periphery of the ligand binding surface of SorCS2 in the published crystal structure of murine SorCS2 in complex with NGF [31]. SorCS2 S484I and R666C both showed reduced capacity to form homodimers. It is likely that both substitutions compromise the structural integrity of one or more domains of SorCS2 which again may compromise dimer formation. In line with this, R666 is localized in close proximity to a disulfide bond in the 10CC module and S484 to a disulfide bond in the β-propeller domain. As the Vps10p domain most likely constitutes the ligand binding surface of most ligands to SorCS2, structural impairment caused by the R702W substitution or other variants will likely compromise ligand binding and thus potentially lead to partial loss-of-function effects.

In the current study, SorCS2 R702W formed receptor complexes with SorCS3, a feature previously described for WT SorCS2 [32]. Though the exact cellular compartment in which SorCS dimerization takes place remains to be elucidated, the apparent retention of SorCS2 R702W in ER leaves us to speculate that the variant could potentially trap WT SorCS receptors in the ER thereby compromising their biological activity. This was supported by our observation that the R702W variant abrogated SorCS2-dependent BDNF signaling in a heterozygous paradigm with WT SorCS2. Dominant negative effects, by ER retention or additional mechanisms, on other SorCS members through heterodimerization would only exist in cells co-expressing SorCS2 with SorCS1 or −3 receptors, a feature fairly common throughout neurons in the human brain [55]. Interestingly, rare deleterious variants in *SORCS3* have been associated with ADHD (albeit at nominal significance) and *SORCS3* SNPs are associated with at least six different neuropsychiatric disorders [4, 26]. Whether SorCS2 R702W could cause dominant negative loss of SorCS3 function and thereby provide another mechanism for risk of psychiatric disorders should be addressed in futures studies.

In conclusion, the present study proposes an important role of SorCS2 perturbations in ADHD and provides a framework for evaluating SorCS2 missense variation.

## Methods

### Plasmids and proteins

The full length SORCS2 expressed sequence is identical to the Genbank acc. no. NP_065828.2; the R702W mutant was created by exchanging a BspEI-EcoNI fragment encoding the R702W variant, the fragment was synthesized by Genscript. The two SORCS2 variants were expressed using the pcDNA3.1/zeo(-) vector (Invitrogen). The cDNA encoding the extracellular domain of human SORCS2 (M1-G1076) and a C-terminal hexaHIS-tag was inserted into the pCpGfree-vitroNmcs vector and transformed into the *E. coli* strain GT115 encoding the *pir* gene (Invivogen). The episomal plasmid was transfected into CHO-K1 cells, stable clones were selected using G418. Cells expressing SORCS2 were subsequently adapted to soluble growth in Hybridoma-SFM medium (Gibco) and expanded in 3-layer flasks (Thermo Fisher Scientific). Recombinant expressed protein was purified from the culture medium using Talon beads (Clontech) according to the manufacturer’s instructions. The propeptide (A20-R143) of BDNF (acc. no. NP_733930.1) was inserted into the pGEX2TK vector (GE Healthcare); The fusion protein was expressed in BL21 cells and purified using glutathione Sepharose beads (GE Healthcare). Plasmids encoding SorCS2 tailless-FLAG and SorCS3 have previously been described [32, 56].

### Size exclusion chromatography

SorCS2 ECD was purified by Ni^2+^ affinity, anion exchange and gel-filtration chromatography. Purified SorCS2 ECD was subjected to analytical gel-filtration using a SD200 increase 3.2/300 (GE Healthcare) in buffer composed of 25 mM Tris pH 7.4 and 300 mM NaCl at a flowrate of 0.04 mL min^−1^. To estimate the hydrodynamic size of SorCS2 ECD, macromolecules of known molecular weight were applied to the size-exclusion column and their elution volume was used to generate a standard curve.

### Cell culturing

The human embryonic kidney (HEK293T) and the Chinese hamster ovary (CHO) cell lines were cultured in DMEM and the neuroblastoma (SH-SY5Y) cell line in DMEM F12 (Lonza) both supplied with 10% FCS (Gibco). Cell lines were evaluated and confirmed free from mycoplasma contamination. Transient transfection was done with fuGENE 6 (Promega) using 3.2 uL/mL fuGENE and 0.8 ug/mL cDNA. Stable cell lines were maintained with 300 μg/mL Zeocin (Invivogen).

### Human iPSC-derived glutamatergic neurons

The human iPSC-derived glutamatergic neurons were obtained from *bit.bio* and maintained according to manufacturer’s protocol. 30.000 neurons per cm^2^ were seeded in Geltrex (ThermoFisher) coated 12 or 96-well polystyrene plates (Thermo Scientific). At day 0–2, neurons were cultured in Neurobasal medium (Merck (Gibco)), containing Glutamax (Merck (Gibco), 25 µM 2-Mercaptoethanol (Thermo Fisher), B27, 10 ng/mL NT3 (R&D Systems), 5 ng/mL BDNF (R&D Systems) and 1 µg/mL doxycycline (Sigma). 10 µM DAPT (Bio-Techne) were additionally introduced from day 2–4. From day 5 and onwards doxycycline and DAPT were removed from media and onwards. Media were changed every 48 h. Human iPSC-derived neurons were transfected at 3 DIV using ViaFect™ Transfection Reagent (Promega) according to manufacturer’s protocol. 0.1 µg or 1 µg of DNA was used for transfection of neurons in 96-well plate or 12-well plate formats, respectively. DNA was mixed with transfection reagent (ViaFect™) at a 1:4 ratio. 48 h post transfection, medium was replaced by maintenance medium. At 14 DIV, neurons were either lysed (12-well plates) in RIPA buffer (50 mM Tris pH 7.4, 150 mM NaCl, 1% Triton X-100, 2 mM EDTA, 0.5% sodium-dexoycholate, 0.1% SDS) supplemented with Pierce protease inhibitor mini cocktail (ThermoFisher) and left for shaking at 300 rpm for 30 min before collection or fixed (96-well plates) in ice cold 4% PFA.

### Shedding, biotinylation, deglycosylation, cross-linking, immunoprecipitation, and pull-down experiments

All experiments were carried out with HEK293T cells transiently or stably expressing SorCS2 unless otherwise stated. Cell lysis was done on ice for 10 min in TNE lysis buffer (20 mM Tris pH 8, 10 mM EDTA, 1% NP40, complete protease inhibitor cocktail (Roche)). Antisera against SorCS2 (F7100), SorCS2 ICD, and SorCS3 were custom made from DAKO. α-SorCS2 (AF4238) were from R&D Systems (used for western blot analysis of iPSC-derived neurons), α-FLAG tag M2 (B3111), and α-tubulin α 4a from Sigma Aldrich, α-insulin receptor b (3025) from Cell Signaling, and α-GST (27-4577-01) from GE Healthcare.

For shedding experiments with galardin, stably expressing cells were washed and cultured in complete medium with or without 30 µM galardin for 24 h. SorCS2 from medium was immunoprecipitated with α-SorCS2 F7100 coupled to Gammabind G-Sepharose beads (Amersham Biosciences). Metabolic labeling of cells was done in poly-L lysine-coated 24-wells by incubating transiently transfected cells in cysteine- and methionine free medium containing 2% dialyzed serum for 20 min. Cells were then incubated 4 h with ~ 300 μCi/mL L-[^35^S]-cysteine and L-[^35^S]-methionine (Pro-mix^TM^, Amersham) and 10 µg/mL brefeldin A in the same medium. Cells were then incubated in complete medium and lysates and culture supernatant collected after various time points. SorCS2 from medium was subsequently immunoprecipitated with α-SorCS2 F7100 and subjected to SDS-PAGE and autoradiography.

For biotinylation studies, stably expressed surface SorCS2 was labeled with 1 mM EZ-Link Sulfo-NHS-Biotin for 2 h at 4 °C according to manufacturer’s protocol (Thermo Scientific). Biotinylated SorCS2 was precipitated by using streptavidin-sepharose beads (GE Healthcare).

Endo H and PNGase F treatment of cell lysates from stably expressing cells were done according to manufacturer’s instructions (NEB).

Dimers of SorCS2 in stably expressing cells were cross-linked with 1.5 mM dithiobis(succinimidyl propionate) (thermoscientific) for 15 min at RT. Cells were lysed in TNE lysis buffer supplemented with 10 mM iodoacetamide (Sigma). Purified SorCS2-hexaHIS in PBS was cross-linked with 0.25 mM dithiobis(succinimidyl propionate) for 15 min at RT. To evaluate dimerization of soluble SorCS2 variants, CHO cells were transiently transfected with soluble SorCS2 constructs and media substituted with HyClone CCM5 culture medium (Thermo Scientific). 48 h post transfection, medium was collected, and primary amines removed by dialysis against PBS. Proteins were cross-linked with 0.25 mM DSP for 15 min. For all dimerization experiments, proteins were separated on a non-reducing NuPage Tris-Acetate 3–8% gradient gel (Invitrogen).

For co-immunoprecipitation studies, lysates from transiently transfected cells were immunoprecipitated ON at 4 °C by use of Gammabind G-Sepharose beads (Amersham) coupled with antibody. Unspecific binding was removed by washing 5 times in DMEM containing 0.05% Tween-20, and proteins eluted by boiling samples in reducing sample buffer.

For pull down studies, cells were transiently transfected with full length SorCS2 variants and lysed 48 h post transfection. SorCS2 was allowed to bind 15 ug GST-BDNFpro ON at 4 °C and the resulting complex subsequently precipitated with glutathione 4B sepharose beads (GE Healthcare) for 3 h at 4 °C. Beads were then washed 5 times with DMEM containing 0.05% Tween-20, and proteins eluted by boiling samples in reducing sample buffer (20 mM DTE, 2.5% SDS).

### Immunofluorescence

HEK293T cells were seeded onto poly-L lysine-coated coverslips 24 h prior to transfection. 24 h post transfection, cells were fixed in 4% ice-cold PFA. HEK293T cells and iPSC-derived neurons were permeabilized with 0.1% Triton-X100 and blocked in 10% FCS. Staining of SorCS2 was done with 1 µg/mL purified α-SorCS2 F7100 (custom made from DAKO, α-SorCS2 (AF4238, R&D Systems), α-calnexin (AB22595, Abcam), α-TGN46 (AHP500G, Biorad), and α-EEA1 (610456, BD Transduction Laboratories). Secondary antibodies were Alexa Fluor-conjugated donkey α-rabbit 488 (A21206, Invitrogen), donkey α-sheep 568, (A21099, Life Technologies), donkey α-goat 568 (A11057, Life Technologies), and donkey α-mouse 568 (A10037, Invitrogen). For neuronal branching assay, images were acquired with a Zeiss LSM780 confocal microscope. For co-localization analyses, images were acquired with a LSM900 inverted laser scanning confocal microscope with Airyscan2 (Zeiss) using a Pln-Apo 63x/1,4 Oil objective. The Airyscan processed images were analyzed using FIJI (ImageJ v. 1.54 f) [57]. Regions of interest were drawn surrounding each transfected cell and colocalization parameters were retrieved using the JACoP (Manders’ coefficients) and Coloc2 (Pearson’s coefficient) plugins [58].

### Sucrose density gradient centrifugation

Preparation and fractionation of postnuclear supernantant from stably expressing HEK293T cells have previously been described [59]. Antibodies for western blotting were the same as described for immunofluorescence. α-cadherin-5 is a polyclonal antibody produced in house.

### ELISA

For quantification of SorCS2 in serum, MaxiSorp plates (Nunc) were coated with 10 µg/mL purified α-SorCS2 F7100 in 0.1 M NaHCO_3_, pH 9.8 for 1 h at 37 °C. After 4 times wash in H_2_O, wells were blocked with 2% BSA (Sigma) in PBS for 1 h at 37 °C. Following 4 times wash in PBS-T, serum samples and standard (purified SorCS2-hexaHIS) were incubated in 2-fold dilution series in PBS-T with 1% BSA over night at 4 °C. Wells were then washed 4 times in PBS-T and incubated with 1 µg/mL α-SorCS2 (R&D, AF4238) for 1 h at 37 °C. After another PBS-T washing step, wells were incubated with HRP-conjugated α-sheep (Dako) for 1 h at 37 °C. After 4 times wash in PBS-T and 4 times in H_2_O, wells were visualized with *o*-Phenylenediamine dihydrochloride (Dako) in 0.1 M citric acid-phosphate buffer pH 5.2 mixed with H_2_O_2_. The reaction was stopped with 0.5 M sulphuric acid and absorbance measured at 490 nM.

### RT-PCR

Stable HEK293T cells were starved in HBSS with 20 mM HEPES pH 7.4 for 6 h and RNA purified using the Nucleospin RNA isolation kit from Macherey-Nagel. mRNA was converted to DNA and amplified with Titanium one-step RT-PCR kit (Clontech). Primer pairs for GRP78 were 5’-CGGTCTACTATGAAGCCCGTCCAGAAA-3’ and 5’-GGAGCAGGAGGAATTCCAGTCAGATCAAA-3’ and for GAPDH 5’-ACCATGGAGAAGGCTGGGGCTCATTT–3’ and 5’–ATGGCATGGACTGTGGTCATGAGTCCTT-3’. Fragments were separated on a 1.5% agarose gel and bands quantified with ImageJ software.

### Neuronal branching assay

*Sorcs2*^-/-^ postnatal day 0 mouse pups of either sex [5] were euthanized and hippocampi dissected into ice-cold Leibowitz’s L-15 medium (Life technologies). Hippocampi were dissociated for 30 min using 20 U/mL pre-activated papain (Bionordica) and washed in DMEM containing 0.01 mg/mL DNase 1 (Sigma) and 10% FCS before being triturated in the same buffer. Following trituration, DMEM was substituted with Neurobasal-A Medium supplemented with B-27 supplement, 2 mM GlutaMAX (all from Gibco), 100 µg/mL primocin (Invivogen), 20 µM floxuridine, and 20 µM uridine (both from Sigma). Cells were then seeded onto pre-coated coverslips (poly-D-lysine and laminin (invivogen)) at a density of 100.000/coverslip and incubated at 37 °C for 24 h. Medium was then substituted with Neurobasal A medium without antibiotics and transfected using lipofectamine (Invivogen). For each co-transfection, 125 ng of plasmid encoding SorCS2 and 125 ng plasmid encoding GFP was added to 100 µl OPTI-MEM reduced serum medium (Gibco) and 0.25 µl plus reagent added to the diluted DNA and incubated at room temperature for 5 min. 1.25 µl lipofectamine LTX was added and the solution incubated at room temperature for 30 min. The DNA-lipid complex solution was then added to neurons and left for 6 h at 37 °C and medium substituted with Neurobasal Amedium with or without 1 nM BDNF (Millipore).

After 72 h, neurons were fixed with 4% PFA and images obtained using confocal microscopy. Branching pattern of GFP positive neurons was analyzed using Zen 2011 image processing (Carl Zeiss). At least 10 neurons were analyzed on 3 or 4 coverslips for each condition.

### Sequencing

#### Norwegian cohort

Norwegian participants were recruited as part of the “ADHD in Norwegian Adults” project. All participants with ADHD and population controls provided signed informed consent, responded to questionnaires and provided blood or saliva samples for biomarker studies [33]. Adult participants with clinically verified ADHD recruited family members who also provided signed informed consents, responded to questionnaires and saliva samples for genetic studies. Targeted capture and massive parallel sequencing were performed at HudsonAlpha Institute for Biotechnology (Huntsville, AL). Genetic variants of interest were validated using Sanger Sequencing as described. DNA from 1846 cases and 7519 controls were also subjected to exome chip analyses. The study was approved by the Regional Committee for Medical Research Ethics of Western Norway (IRB 00001872, project 2013/543).

#### German and Dutch cohorts

We examined adult subjects (age > 18 years) clinically diagnosed with persistent ADHD in accordance with DSM-IV guidelines, excluding those with an intellectual disability. The participants were part of a larger cohort assembled under the umbrella of the International Multicenter persistent ADHD Collaboration (IMpACT), utilizing two primary sites for recruitment: Radboud University in the Netherlands, and the University of Würzburg in Germany. Our control sample included subjects from the IMpACT cohort at Radboud University and an additional 1766 German individuals. The German controls were recruited from the German MI Family Study [60] and the Angio-Lub study and have been whole-exome sequenced by the MIGen Exome Sequencing Consortium: Lubeck Heart Study (dbGAP accession number phs000990/DS-CVD). Among the dbGAP controls, 870 individuals were identified with cardiovascular conditions and 896 were without.

Biological samples collected at the Dutch and German IMpACT sites were exome-sequenced at BGI, Shenzhen, China, using their exome-capture kit targeting 58.8 Mb, and pair-end sequenced to an average 50x depth on the Illumina HiSeq2000 platform. The exome data from dbGAP was generated at the Broad Institute of Harvard and MIT using Illumina’s ICE Capture reagent kit and sequencing on an Illumina HiSeq 2000 or 2500. All bam files of clinical samples and control samples underwent a standardized QC process in line with prior protocols [46]. Post-QC, the dataset comprised 1078 ADHD cases and 1738 controls. Further analysis was restricted to variants that were singletons in 2816 samples and absent from the non-psychiatric subset of individuals in the ExAC [61] database. Filtering was not based on gnomAD [29, 62] due to the inclusion of dbGAP control data. Note, the R666C mutant was from an ADHD patient that was subsequently excluded from this cohort due to genetic background stratification but was included in the manuscript due to its close structural proximity to R702.

#### Missense variant deleteriousness predictions

The variants from the two cohorts were annotated using the VEP database to obtain SIFT [34], Polyphen-2 [35], CADD-PHRED [36], MutationTaster [37], AlphaMissense [38] and MPC [39] scores. For each variant, a consensus score of the predictions was obtained by adding one point for each if the predictor annotated the variant deleterious or damaging (SIFT, PolyPhen-2, MutationTaster and AlphaMissense) or the value prediction was above a certain threshold (MPC > 2, CADD-PHRED > 20). For burden analysis of damaging missense variants, we used variants with consensus scores ≥3 in Fisher’s exact test. Consensus scores and statistics were calculated using R version 4.3.1.

### Magnetic resonance imaging (MRI)

MRI scans of Norwegian adult control persons and ADHD cases were obtained using a 3.0 Tesla GE-Signa system (General Electric Medical Systems, Milwaukee, WI) with an eight-channel headcoil. T1-weighted images were acquired using a Fast Spoiled Gradient Recall sequence (FSPGR, echo time = 14 ms, repetition time = 400 ms, inversion time = 500 ms) collecting 188 sagittal slices of 1 mm thickness (no interslice gap; scan matrix: 256 × 256; field of view of 256 × 256 mm) to achieve a reconstructed voxel size of 1 × 1 × 1 mm [28, 63].

### Ethics statement

The studies involving Norwegian human participants were reviewed and approved by the Regional Committee for Medical Research Ethics of Western Norway (IRB 00001872, project 2013/543). Informed consent to participate was obtained from all participants.

Our study protocol, ethical approval and informed consent state that we will perform DNA genotyping and sequencing of all participants, but that we cannot share individual genetic information with any study participant. For MRI studies, all images were screened by a neuroradiologist, and participants were contacted in cases where significant pathology was detected. We have not reached out to the participants to ask for permission to publish specific brain images. In this case, this would imply that we also shared the genotype information for all the family members shown in the pedigree in Fig. 1A. The MRI images shown in Fig. 1C are not immediately identifiable. However, we know that re-identification of anonymized MRI head images may be possible with publicly available software [64].

All presented methods were performed in accordance with relevant guidelines and regulations.

## Supplementary information

Supplementary Figure 1

Supplementary Figure 2

Supplementary Figure 3

Supplementary Figure 4

Supplementary Table 1

## Data Availability

The datasets generated and/or analysed during the current study are available from the corresponding author on reasonable request.
