# Supplementary figures and images for: A low frequency damaging SORCS2 variant identified in a family with ADHD compromises receptor stability and quenches activity
Source: Mol Psychiatry. 2025 Sep 18;31(3):1311–24. doi: 10.1038/s41380-025-03242-3 (PMC12916480; doi:10.1038/s41380-025-03242-3)

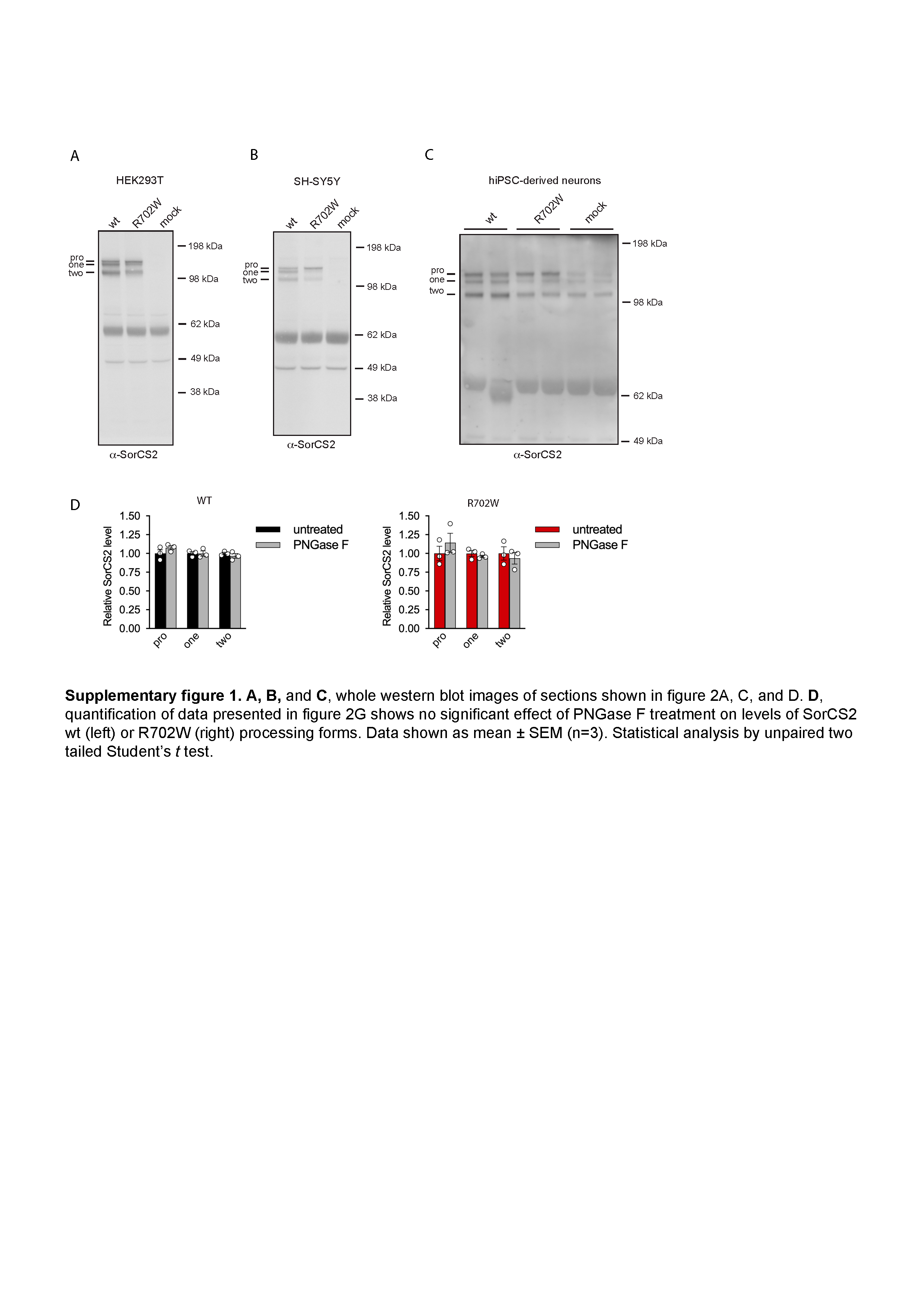

Supplement: Supplementary file 1 — Supplementary Figure 1 [file 41380_2025_3242_MOESM1_ESM.tif]

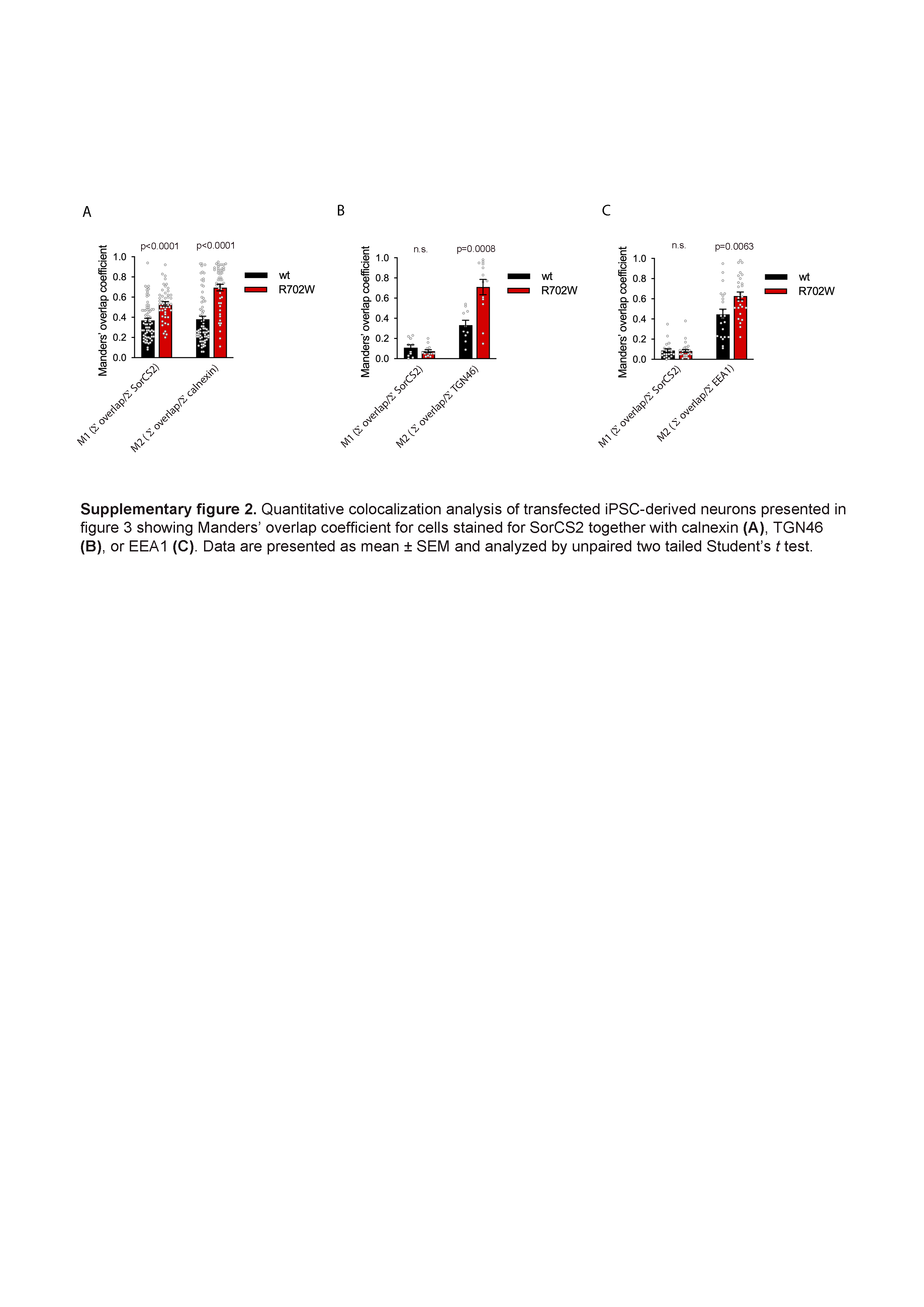

Supplement: Supplementary file 2 — Supplementary Figure 2 [file 41380_2025_3242_MOESM2_ESM.tif]

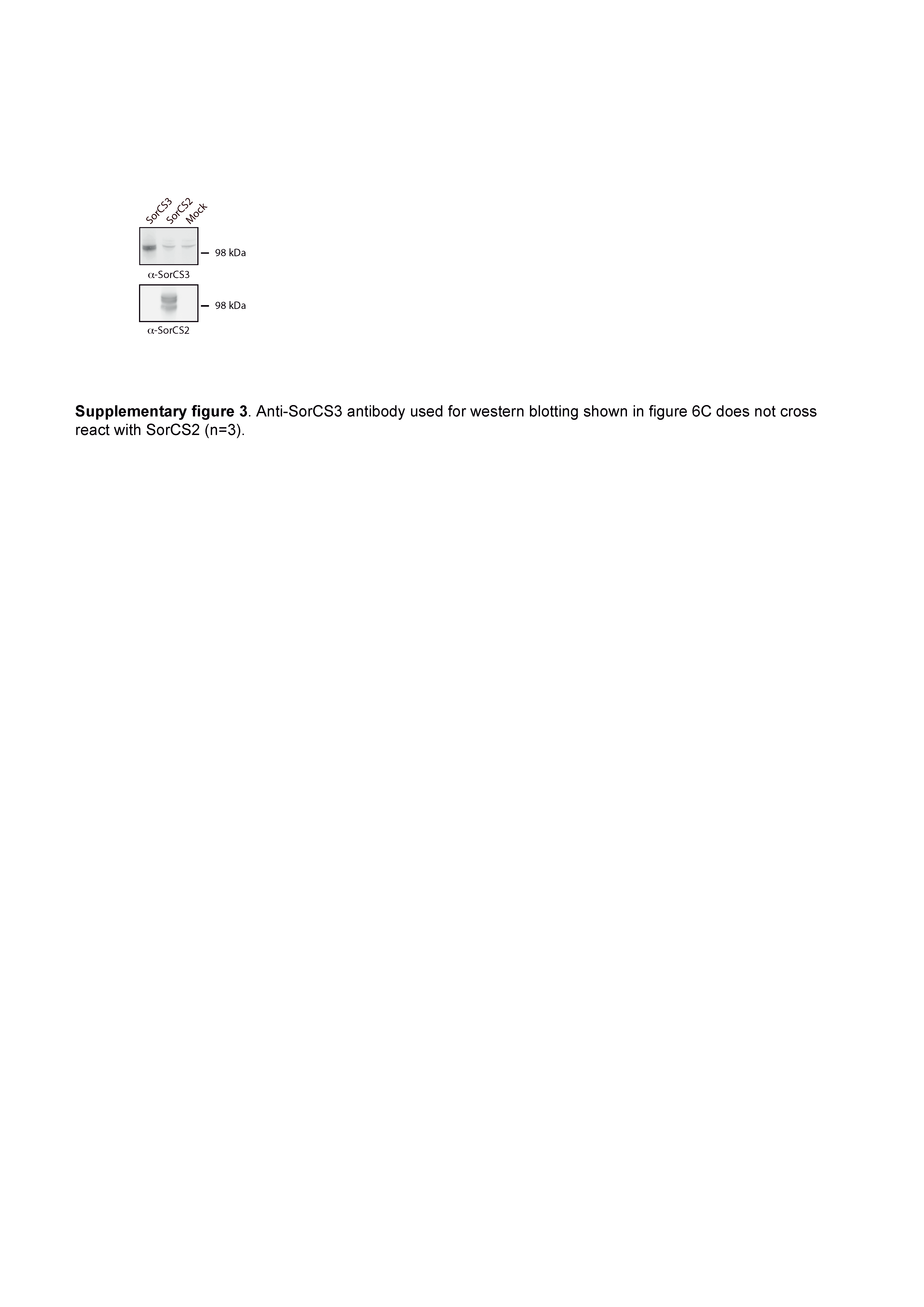

Supplement: Supplementary file 3 — Supplementary Figure 3 [file 41380_2025_3242_MOESM3_ESM.tif]
